# Linderalactone Suppresses Pancreatic Cancer Development *In Vitro* and *In Vivo* via Negatively Regulating PI3K/AKT Signaling Pathway

**DOI:** 10.1155/2022/8675096

**Published:** 2022-08-04

**Authors:** Dongchao Xu, Mengyao Tian, Wangyang Chen, Ying Bian, Xiaofeng Xia, Qiang Liu, Liyun Zheng, Xiaofeng Zhang, Hongzhang Shen

**Affiliations:** ^1^Department of Gastroenterology, Affiliated Hangzhou First People's Hospital, Zhejiang University School of Medicine, Hangzhou 310000, China; ^2^Hangzhou Institute of Digestive Diseases, Hangzhou 310000, China; ^3^Key Laboratory of Integrated Traditional Chinese and Western Medicine for Biliary and Pancreatic Diseases of Zhejiang Province, Hangzhou 310000, China

## Abstract

Linderalactone is one of the main extracts of Linderae Radix, which is widely used in traditional Chinese medicine. There have been few studies on the antitumor effect of linderalactone in the past. In this study, we explored the anti-pancreatic cancer activity of linderalactone *in vitro* and *in vivo*. The  results showed that linderalactone inhibited the proliferation of pancreatic cancer cells in a time- and dose-dependent manner. Cell migration and invasion were significantly inhibited by linderalactone. The cell cycle was arrested in the G2/M phase, and the expression levels of cell cycle-associated proteins changed significantly with linderalactone treatment. In addition, linderalactone induced cell apoptosis and altered the expression of apoptotic markers, such as caspase 3 and PARP1. Mechanistically, linderalactone suppressed the PI3K/AKT  signaling pathway by downregulating the phosphorylation of PI3K and AKT. The xenograft study results were consistent with the *in vitro* results, and there was no obvious chemical toxicity. Thus, our research demonstrated that linderalactone exhibits antitumor activity against pancreatic cancer and may be developed as a potential anti-pancreatic cancer agent in the future.

## 1. Introduction

The incidence of pancreatic cancer, a highly fatal solid tumor, has increased in recent years, and the mortality rate is extremely high: only 10% of patients survive for more than 5 years [1, 2]. Due to the lack of effective screening methods, more than 90% of patients have metastatic disease upon diagnosis, which is the main reason for the high mortality of pancreatic cancer [3]. For most patients with distant metastases, chemotherapy is the only treatment strategy, but the currently used chemotherapeutics have a very limited ability to prolong patient survival [4]. Currently, chemotherapy drugs, such as gemcitabine, erlotinib, nab-paclitaxel, and 5-fluorouracil, are widely used to treat pancreatic cancer [5]. However, adverse side effects and drug resistance significantly limit their clinical application [6]. Therefore, there is an urgent need to develop novel antitumor agents.

Natural plant products are now receiving much attention for the treatment of tumors. For example, the combination of paclitaxel, a secondary metabolite purified from yew, with gemcitabine has become a typical treatment for pancreatic cancer [7, 8]. Here, we explored a compound extracted from Linderae Radix that has the potential to treat pancreatic cancer. Linderae Radix has been used for hundreds of years as a traditional Chinese herbal plant with tremendous medicinal properties. It is widely used in the treatment of gastrointestinal diseases, mainly for abdominal distension and pain, nausea, and vomiting. Although unprecedented accomplishments have been achieved with modern medicine, Linderae Radix is used in clinical practice because it exerts analgesic and anti-inflammatory effects with an excellent curative effect and few side effects [9].

Linderalactone is a hydroxylated biphenyl compound isolated from Linderae Radix that has anti-inflammatory properties [10, 11]. Previous studies have found that linderalactone has value in the treatment of lung cancer [12]. In light of the extensive role of Linderae Radix in digestive system diseases and its antitumor effect in lung cancer, we have a keen interest in linderalactone and thus explored whether it inhibits the activity of pancreatic cancer. In this study, we showed the cytotoxicity of linderalactone in pancreatic cancer *in vitro* and its inhibitory effect on tumor progression *in vivo*. This study revealed for the first time that linderalactone plays a role in pancreatic cancer by inhibiting the PI3K/AKT signaling pathway.

## 2. Materials and Methods

### 2.1. Cell Culture and Reagents

Four pancreatic cancer cell lines, ASPC-1, BXPC-3, CFPAC-1, and SW-1990 were obtained from American Type Culture Collection (ATCC, USA). All cell lines were cultured in the RPMI-1640 medium (Sigma, USA) supplemented with 10% fetal bovine serum (Gibco, USA) and a 1% penicillin and streptomycin mixture (Solarbio, China). The cells were placed in a cell incubator (Thermo Scientific, USA) with 37°C and 5% CO_2_. The Luciferase Mycoplasma Detection Kit (TransGen Biotech, China) was used to confirm that the cells were free of mycoplasma contamination. Linderalactone (Chengdu Herbpurify, China) was dissolved in dimethyl sulfoxide (DMSO, Sigma, USA).

### 2.2. Cell Viability

The Cell Counting Kit-8 (CCK-8, Bimake, USA) was used to detect cell viability. In brief, pancreatic cancer cells were plated at 3000 cells per well in 96-well plates and treated with varying concentrations of linderalactone (0, 30, 40, 50, 60, 70, 80, 90, and 100 *μ*M) for different periods of time (0, 24, and 48 h). Ten microliters of CCK-8 solution were added to each well, and the plates (Corning, USA) were incubated at 37°C for 2 h. The percentage of surviving cells was calculated by measuring the absorbance at an optical density of 450 nm using a microplate reader (Thermo Fisher, USA). The IC50 value was determined based on a linear regression curve.

### 2.3. EdU Assay

The EdU Cell Proliferation Kit with Alexa Fluor 488 was purchased from Beyotime (China). All steps were conducted in accordance with the manufacturer's instructions. In brief, pancreatic cancer cells were treated with varying concentrations of linderalactone, and then, EdU was added and incubated with the cells for one-tenth of the cell doubling time (BXPC-3, 5 h; CFPAC-1, 3 h). The cells were incubated with Hoechst stain for 20 min to visualize the nucleus and then examined under a fluorescence microscope (Nikon, Japan).

### 2.4. Colony Formation Assay

Pancreatic cancer cells were seeded into six-well plates at a density of 1 × 10^3^ cells/well. Three days later, linderalactone was added to the medium at varying concentrations and the cells were cultured for 24 h. After 7 days, the cells were washed with PBS (Solarbio, China) and fixed with 4% paraformaldehyde (PFA, Beyotime, China) for 30 min. Subsequently, 1% crystal violet solution (Beyotime, China) was used to stain the colonies. After 10 min, the plates were washed with pure water for further counting and analysis.

### 2.5. Wound-Healing Assay

Pancreatic cancer cells (1 × 10^6^) were seeded into six-well plates and cultured to a confluence greater than 90%. The medium was replaced with a serum-free medium for 24 h to eliminate the effect of cell proliferation on the experimental results. The confluent monolayer of cells was scraped with a 10 *μ*L sterile pipette tip, and the cells were then incubated with a serum-free medium containing varying concentrations of linderalactone. The scratch width was recorded using phase-contrast microscopy at 0 h and 24 h. The migration distance was calculated using ImageJ software (National Institutes of Health, USA).

### 2.6. Transwell Assay

Transwell assays were used to evaluate migration and invasion. For migration, pancreatic cancer cells (5 × 10^4^) were mixed with varying concentrations of linderalactone in a serum-free medium. Transwell cell culture chambers (Falcon, USA) with 8.0 *μ*m Transparent PET Membranes were used. Two hundred microliters of a cell-medium mixture was placed in the upper chamber, and 600 *μ*L of medium containing 20% FBS was placed in the lower chamber as a cell migration chemoattractant. After 24 h of incubation in the incubator, the cells in the upper chamber were removed with cotton swabs and the cells in the lower chamber were fixed in 4% PFA for 30 min at room temperature and stained with 1% crystal violet solution for 20 min at room temperature. After the cells were dried, the number of stained cells was counted. For the invasion assays, all the steps were the same as those for the migration assays, except that the 8.0 *μ*m transparent PET membranes were coated with a 0.5 mg/mL basement membrane matrix (Corning, USA).

### 2.7. Cell Cycle Analysis

A cell cycle analysis kit was purchased from Beyotime (China). In brief, pancreatic cancer cells (9 × 10^4^ per well) were seeded in 6-well plates. After overnight culture, the cells were treated with varying concentrations of linderalactone. After 24 h of treatment, the cells were collected and ﬁxed with prechilled 70% alcohol for 12 h. Then, the cells were washed with PBS; resuspended in a PBS mixture containing 0.05% Triton X-100, 0.1 mg/mL DNase-free RNase A, and 25 *μ*g/mL PI; and incubated for 30 min at 37°C in the dark. A flow cytometer (BD Biosciences, USA) was used to detect light scatter and red fluorescence at an excitation wavelength of 488 nm.

### 2.8. Cell Apoptosis Analysis

The FITC Annexin V/Dead Cell Apoptosis Kit was purchased from Invitrogen (USA). Pancreatic cancer cells (9 × 10^4^ per well) were treated with varying concentrations of linderalactone for 24 h. The cells were then washed with prechilled PBS and centrifuged, and the supernatant was removed. The cells were resuspended in annexin binding buffer, and 5 *μ*L of FITC annexin V and 1 *μ*L of 100 *μ*g/mL PI working solution were added to each 100 *μ*L aliquot of cell suspension. After incubating the cells at room temperature for 15 min, annexin binding buffer was added, and the stained cells were analyzed by flow cytometry with fluorescence emission measured at 530 nm.

### 2.9. Western Blotting

Western blotting was performed as previously described [13]. The cells were collected and lysed in RIPA lysis buffer (Fude, China) with protease inhibitors (Thermo Scientific, USA). Protein (50 *μ*g) was separated by SDS-PAGE (Sangon, China) and transferred to PVDF membranes (Millipore, USA). After blocking with 3% BSA (Solarbio, China), the membranes were incubated with primary antibodies overnight. The next day, the membranes were washed with TBST (Sangon, China) and incubated with the secondary antibody (Promega, USA) for 90 min at room temperature. Enhanced chemiluminescence detection (Fude, China) was used to detect the target protein. Primary antibodies against the following proteins were used: p-PI3K, PI3K, p-AKT, AKT, PCNA, Ki-67, caspase 3, PARP1, Bcl-2, Bax, cyclin A2, cyclin B1, cyclin D1, and cyclin E1. All antibodies were purchased from Abcam (USA).

### 2.10. Xenograft Experiments

Four- to six-week-old BALB/c nude mice were purchased from Shanghai SLAC Laboratory Animal Company (China). The protocol was approved according to the ethical standards of the Institutional Animal Care and Use Committee of Zhejiang Chinese Medical University and complied with the Regulations for the Administration of Affairs Concerning Experimental Animals (approved by the State Council of China, No. SYXK (Zhejiang) 2018-0012). The pancreatic cancer cell line BXPC-3 was injected into the right abdomen of each nude mouse. After two weeks, 18 nude mice with tumors of the same size were selected into three groups. Normal saline or low-dose (25 mg/kg) or high-dose (50 mg/kg) linderalactone was injected every three days, and tumor weight and volume were measured. After three weeks, the mice were sacrificed, and the tumors were removed. The tumor volume was calculated as 0.5 × lengths × width^2^.

### 2.11. Statistical Analyses

All experiments were repeated at least three times to ensure the reliability of the results. Data were analyzed using SPSS 12.0, and the results are expressed as the mean ± standard error. An unpaired Student's *t*-test was used to determine statistical significance, which was indicated by *p* < 0.05.

## 3. Results

### 3.1. Linderalactone Inhibits the Viability of Pancreatic Cancer Cells

**Figure 1:**
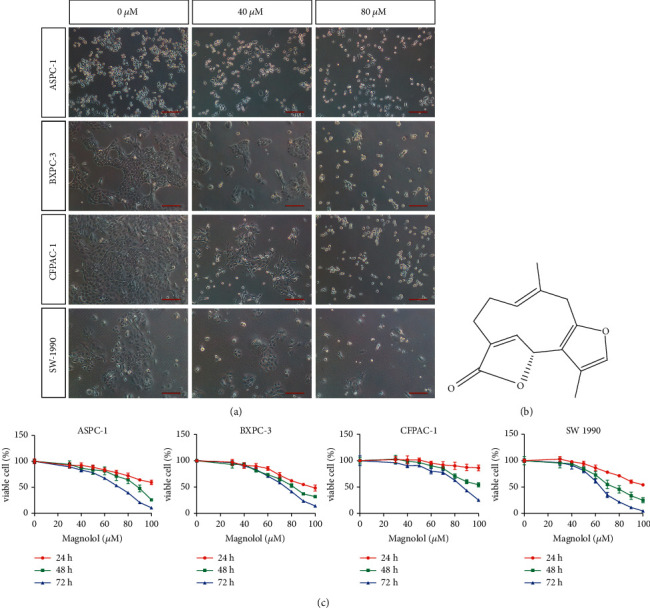
Linderalactone inhibits the viability of pancreatic cancer cells. (a) Morphological changes of pancreatic cancer cells exposed to LL. (b) The chemical structure of LL. (c) CCK8 assay detected the viability of pancreatic cancer cells under varying concentrations and time of LL treatment. Scale bar = 200 *μ*m.

**Figure 2:**
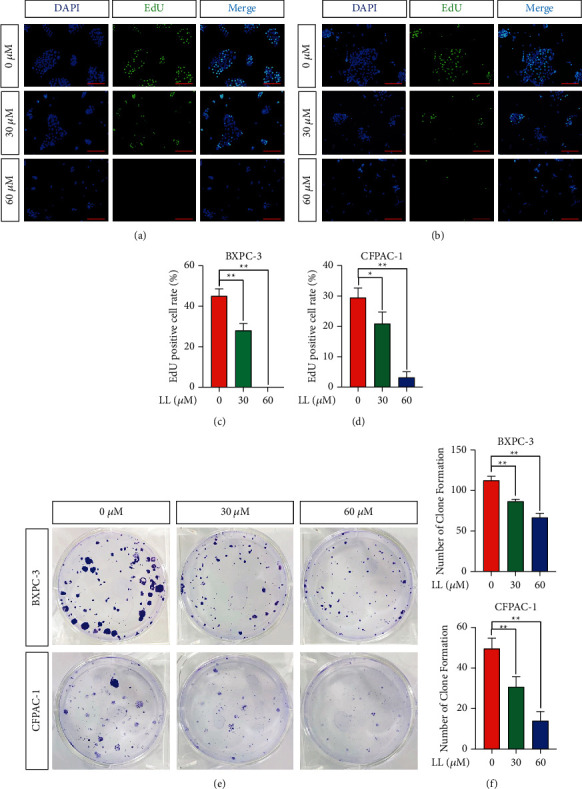
Linderalactone inhibits the proliferation of pancreatic cancer cells. (a, b) EdU staining assay detected the proliferation of BXPC-3 and CFPAC-1 cell lines under varying concentrations of LL treatment after 24 hours. (c, d) Quantiﬁcation of EdU stain: the percentage of EdU positive cells to the total number of cells. (e) Clone formation assay to detect the effect of LL on BXPC-3 and CFPAC-1 cell lines. (f) Quantification of clone formation. Scale bar = 200 *μ*m. ^*∗*^*p* < 0.05; ^*∗∗*^*p* < 0.01.

To determine the effect of linderalactone on pancreatic cancer, we treated four pancreatic cancer cell lines with increasing concentrations of linderalactone. After 72 h of treatment with linderalactone, all tumor cell lines underwent quantitative and morphological changes. As shown in Figure 1(a), the cells lost their unique morphology, appeared round, and showed worse adherence to the well.  Figure 2 shows the chemical structure of linderalactone. Given the inhibitory effect of linderalactone on pancreatic cancer cells, we performed CCK-8 assays to detect the activity of cells treated with varying concentrations of linderalactone for different periods of time. The results are shown in Figure 1(c). Linderalactone inhibited pancreatic cancer activity in a time- and dose-dependent manner.

### 3.2. Linderalactone Inhibits the Proliferation of Pancreatic Cancer Cells

Replicative immortality is one of the hallmarks of cancer [14]. Cell proliferation is accompanied by DNA replication. We performed EdU assays to determine the effect of linderalactone on cell proliferation. The results showed that (Figures 2(a)–2(d), Supplementary Figures 1(a)–1(d)) the population of EdU-positive cells decreased as the concentration of linderalactone increased compared with the control. Colony formation experiments were conducted to evaluate the long-term effects of linderalactone on pancreatic cancer cells. The number and size of colonies were significantly reduced by treatment with linderalactone (Figures 2(e) and 2(f)). These results indicated that linderalactone can inhibit the proliferative potential of pancreatic cancer.

### 3.3. Linderalactone Inhibits the Migration and Invasion of Pancreatic Cancer Cells

**Figure 3:**
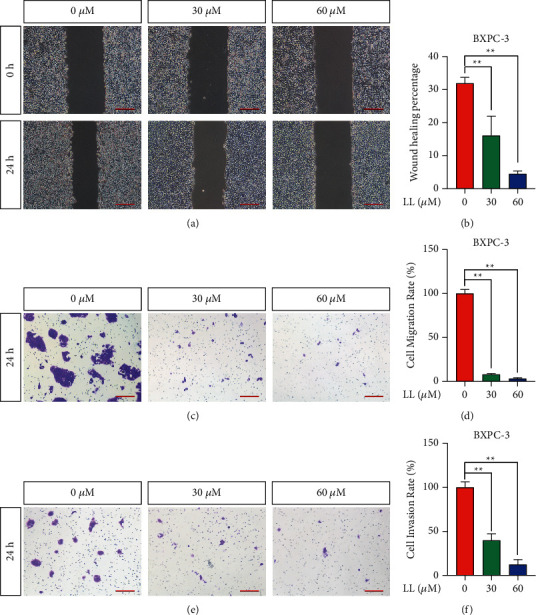
Linderalactone inhibits the migration and invasion of pancreatic cancer cells. (a) Wound-healing assay detects the migration ability of BXPC-3 under varying concentrations of LL after 24 hours. (b) Quantiﬁcation of wound-healing assay: the area of cell migration in 24 hours accounts for the percentage of the wound area in 0 hours. (c) The transwell assay detected the migration ability of cells under varying concentrations of LL treatment after 24 hours. (d) Quantiﬁcation of transwell assay: percentage of the compound treatment group in the control group. (e) The transwell assay with Matrigel to detect cell invasion ability under varying concentrations of LL treatment after 24 hours. (f) Quantiﬁcation of transwell assay: percentage of the compound treatment group in the control group. Scale bar = 200 *μ*m. ^*∗*^*p* < 0.05; ^*∗∗*^*p* < 0.01.

The systemic metastasis of tumors often underlies the inability to cure the disease [15]. We used wound-healing and Transwell experiments to evaluate the migration ability of cells. In addition, Transwell assays with Matrigel were used to evaluate cell invasion ability. To reduce the influence of cell proliferation on the migration results in the scratch assays, we first starved the cells with a serum-free medium for 24 h; thus, the results only reflected the effects of linderalactone. As shown in Figures 3(a) and 3(b), the migration distance of cells treated with linderalactone was significantly reduced and the distance migrated by cells in the 60 *μ*M group was shorter than that by those in the 30 *μ*M group. Transwell experiments were used to detect the migration ability of cells in 3D space, and the results (Figures 3(c) and 3(d)) were consistent with the scratch assay results. As the concentration of linderalactone increased, the migration ability decreased. Cell invasiveness was evaluated using Transwell assays with Matrigel (Figures 3(e) and 3(f)). In general, linderalactone not only inhibited proliferation but also showed effective antimetastatic activity. All the results shown in Figure 3 were from experiments performed with BXPC-3 cells, and these experiments were repeated in CFPAC-1 cells (Supplementary Figure 2).

### 3.4. Linderalactone Arrests the Cell Cycle and Induces Apoptosis

**Figure 4:**
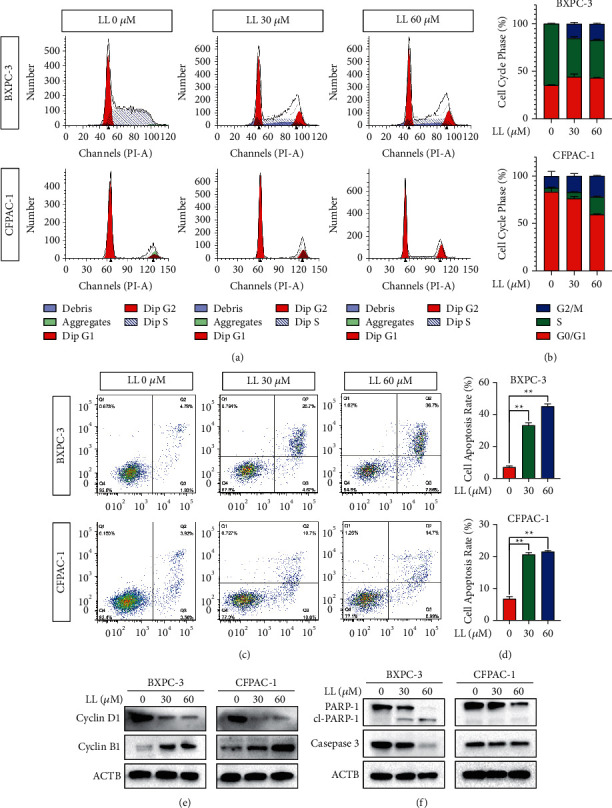
Linderalactone blocks the cell cycle and induces apoptosis. (a) Flow cytometry using PI staining to detect the cycle distribution of BXPC-3 and CFPAC-1 cell lines treated with varying concentrations of LL for 24 hours. (b) The percentage of cell cycle distribution for BXPC-3 and CFPAC-1. (c) Annexin V/PI staining to detect the apoptotic death of BXPC-3 and CFPAC-1. (d) The percentage of cell apoptosis for BXPC-3 and CFPAC-1. (e, f) Western blot analysis of key cell cycle and cell apoptosis markers. ^*∗*^*p* < 0.05; ^*∗∗*^*p* < 0.01.

Our previous research found that linderalactone inhibits the proliferation of pancreatic cancer cells, so we used flow cytometry to detect potential changes in the cell cycle. The results are shown in Figures 4(a) and 4(b). After treatment with linderalactone, the number of cells in the G2/M phase increased significantly compared with that in the control group. Western blotting was used to detect the expression of cyclin A2, cyclin B1, cyclin D1, and cyclin E1. The protein levels of cyclin B1, which marks the G2/M phase, increased significantly, while those of cyclin A2, cyclin D1, and cyclin E1, which marks the G1 or S phase, decreased, confirming the cell cycle results obtained by flow cytometry (Figure 4(e)). Cell cycle arrest is often accompanied by apoptosis, so we subsequently detected apoptosis by flow cytometry, and the results were consistent with our expectations (Figures 4(c) and 4(d)): linderalactone induced apoptosis. The expression levels of Bcl-2, Bax, PARP1, and caspase 3 were determined (Figure 4(f)); the decrease in Bcl-2, PARP1, and caspase 3 protein levels and the increase in Bax and cleaved PARP1 levels indicated the occurrence of apoptosis.

### 3.5. Linderalactone Exhibits Antitumor Activity by Inhibiting the PI3K/AKT Signaling Pathway

**Figure 5:**
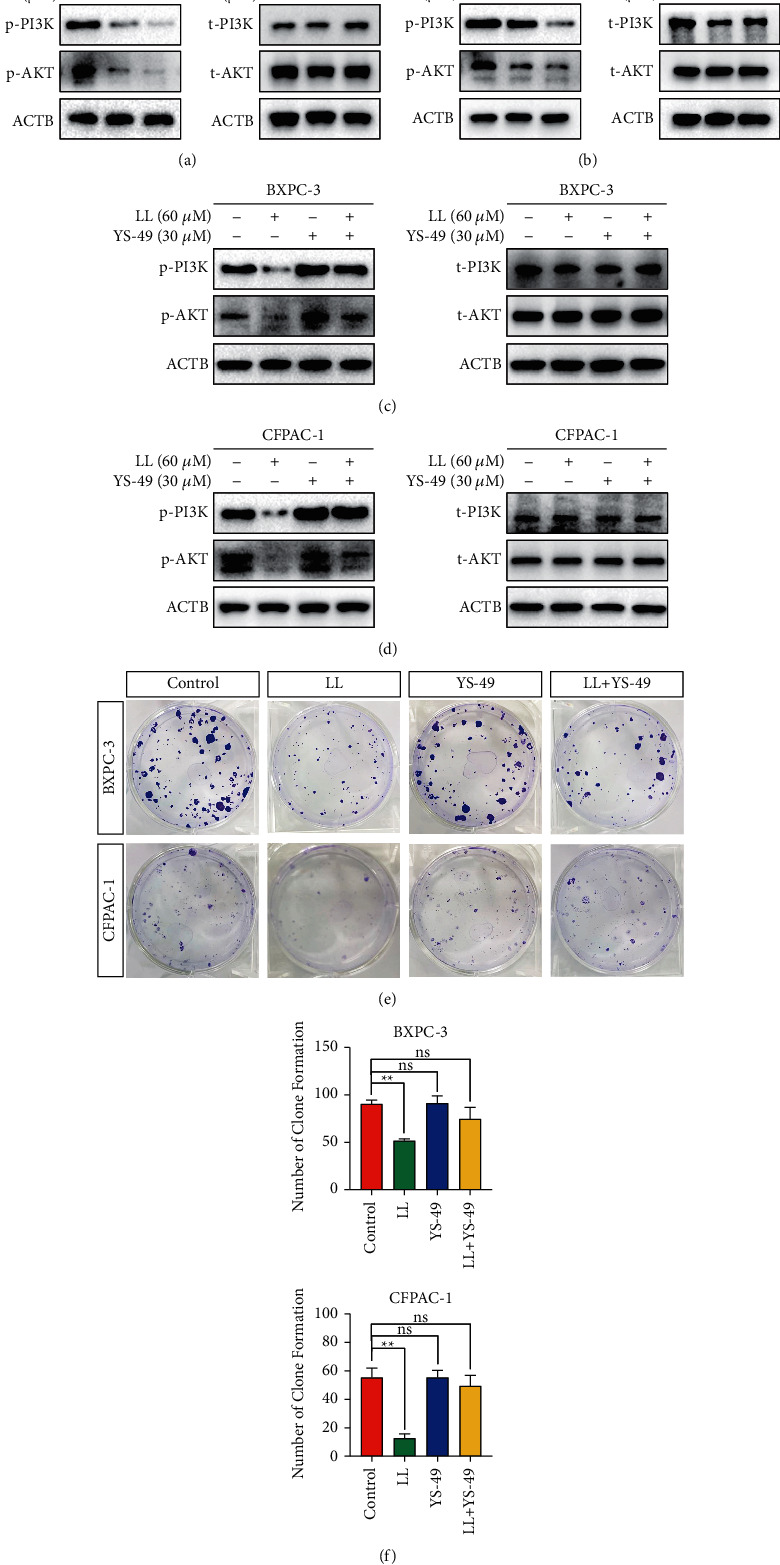
Linderalactone exhibits antitumor activity by inhibiting the PI3K/AKT signaling pathway. (a, b) Western blot analysis of phosphorylated PI3K and phosphorylated AKT, total PI3K, and total AKT expression in BXPC-3 and CFPAC-1 cells after treatment with varying concentrations of LL. (c, d) After LL or YS-49 treatment, the expression of phosphorylated PI3K and phosphorylated AKT and total PI3K and total AKT in BXPC-3 and CFPAC-1 cells. (e) After LL or YS-49 treatment, the clone formation assay detects the proliferation ability of BXPC-3 and CFPAC-1. (f) Quantification of clone formation. ^*∗*^*p* < 0.05; ^*∗∗*^*p* < 0.01.

We next tried to elucidate how linderalactone inhibits pancreatic cancer activity. It is well known that pancreatic cancer is a malignant tumor driven by oncogenes. Numerous studies have found that genes such as K-ras, Smad, and Stat are active in pancreatic cancer. We evaluated the changes in a number of signaling pathway components, including PI3K/AKT, before and after linderalactone treatment. Significant changes in the PI3K/AKT signaling pathway were observed after treatment (Figures 5(a) and 5(b)). Previous studies have found that inhibiting the PI3K/AKT signaling pathway can effectively inhibit the function of pancreatic cancer; thus, we speculated that linderalactone functions in this way [16, 17]. To confirm our conjecture, we used YS-49, a PI3K/AKT activator [18]. As shown in Figures 5(c) and 5(d), linderalactone alone decreased the levels of p-PI3K and p-AKT, while YS-49 alone did not affect the phosphorylation of these proteins. When the two compounds were combined, YS-49 reversed the inhibitory effect of linderalactone on p-PI3K and p-AKT. None of the evaluated treatments caused changes in total PI3K and AKT levels. In the colony formation assays, cotreatment with YS-49 effectively reversed the antitumor effects of linderalactone. Therefore, we confirmed that linderalactone inhibits the tumor activity of pancreatic cancer by inhibiting PI3K/AKT.

### 3.6. Linderalactone Inhibits Tumor Progression *In Vivo*

**Figure 6:**
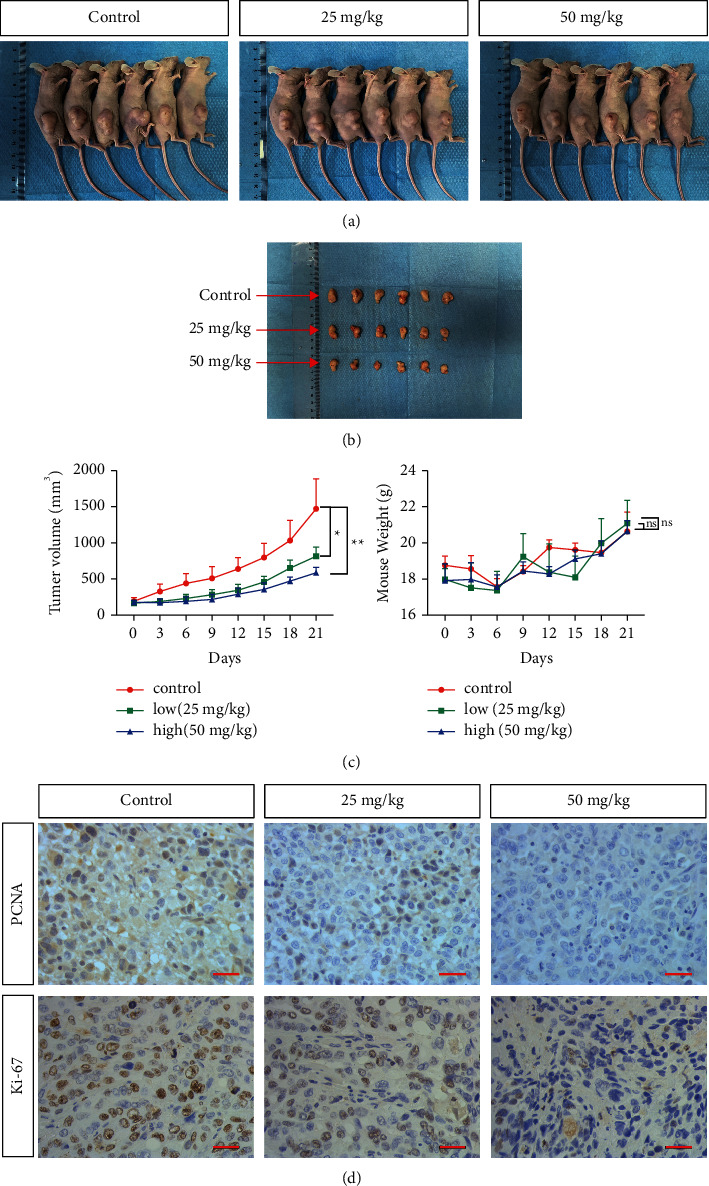
Linderalactone inhibits tumor progression *in vivo*. (a) Images of the control group and low-dose (LL 25 mg/kg) and high-dose (LL 50 mg/kg) tumor-bearing mice. (b) Quantitative volume of the tumor progression: the tumor volume was measured every three days for three weeks after the injection of LL. (c) Quantitative mice weight: the body weight of the mice was measured every three days for three weeks after the injection of LL. (d) Immunohistochemical detection of PCNA and Ki-67 expression level in tumor cells. ^*∗*^*p* < 0.05; ^*∗∗*^*p* < 0.01.

To explore the effect of linderalactone on tumor growth *in vivo*, a nude mouse pancreatic tumor model was constructed. Physiological saline or low-dose (25 mg/kg) linderalactone or high-dose (50 mg/kg) linderalactone was injected into the abdominal cavity of the mice once every three days, and tumor volume and weight were recorded. The experimental results revealed that the tumors of mice treated with linderalactone were generally smaller than those of mice in the control group and the therapeutic effect of high-dose linderalactone was better than that of low-dose linderalactone (Figures 6(a) and 6(b)). In addition, trends in the body weight of mice treated with linderalactone were similar to those of mice in the control group (Figure 6(c)), with no significant difference, indicating that linderalactone does not exert significant toxicity. The immunohistochemistry results (Figure 6(d)) showed that PCNA and Ki-67 expressions were lower in the experimental group than in the control group. In general, the antitumor activity of linderalactone *in vivo* was consistent with that *in vitro*.

## 4. Discussion

Although some progress has been made in prolonging patient survival, adverse reactions to chemotherapy drugs used to treat pancreatic cancer have seriously affected patients' quality of life. Phytochemicals are generally considered to have minor adverse effects and great potential in the treatment of tumors. Phytochemicals are a class of biological secondary metabolites that are abundant in fruits, grains, and vegetables. In recent years, the role of phytochemicals as anticancer agents has been increasingly elucidated, as studies have shown that they induce apoptosis, inhibit proliferation, and increase chemical sensitivity [19]. Previous studies have shown that lutein can reduce the levels of K-ras and AKT in mouse colon cancer and induce cell cycle arrest [20]. Lycopene can be used as a preventive agent because it decreases MMP-7 expression and inhibits the invasion of HT-29 human colon cancer cells [21]. Procyanidin therapy exerts antiproliferative and anti-invasive effects in pancreatic cancer cells and is an effective chemopreventive agent for this disease [22].

Linderae Radix has been used in East Asian countries such as China and Japan for centuries as a folk medicine without any obvious toxicity. Linderae Radix has anti-inflammatory and antioxidant effects in the treatment of alcoholic liver injury and hyperlipidemia [23–26]. In this study, we explored the antitumor effects of linderalactone, an extract of Linderae Radix, on pancreatic cancer. We used four pancreatic cancer cell lines and mouse tumor-bearing models for *in vitro* and *in vivo* studies. The antitumor activity of linderalactone was evaluated, and its mechanism of action was investigated [27–30].

The inhibition of proliferation and metastasis and the induction of cell cycle arrest are effective means of treating tumors. Here, our study found that linderalactone effectively inhibited the proliferation of pancreatic cancer cells both in the short and long term. Invasion and migration are hallmarks of cancer cell metastasis, and our research showed that linderalactone effectively inhibited the migration and invasion of pancreatic cancer cells, thereby inhibiting metastasis. Flow cytometry analyses revealed that pancreatic cancer cells arrested the G2/M phase of the cell cycle in response to linderalactone treatment and apoptosis occurred. Cell cycle arrest in the G2/M phase stops tetraploid cells from dividing. Numerous drugs that induce tumor cell arrest in the G2/M phase have been used for tumor treatment. The G2/M checkpoint is one of the key targets of tumor treatment. The cell cycle is regulated by cyclin B1. Our results showed that cyclin B1 expression decreased, confirming our flow cytometry results. In addition, our apoptosis analyses and western blot experiments proved that linderalactone induced the apoptosis of pancreatic cancer cells by activating the apoptosis pathway. It will perhaps be more appropriate to further explore the effects of linderalactone on pancreatic cancer metastasis in an orthotopic mouse model.

Pancreatic cancer is known to be driven by gene mutations. Most pancreatic cancers have TP53 gene mutations leading to the loss of TP53. This leads to the upregulation of the PI3K/AKT signaling pathway. The PI3K/Akt pathway is one of the most commonly deregulated signaling pathways in pancreatic cancer [31]. PI3K is activated in response to various extracellular signals, such as antigens, epidermal growth factor receptors (EGFRs), and cytokines. AKT is a conserved serine protein kinase in the AGC subfamily of protein kinases [32]. Many studies have shown that AKT regulates important downstream effectors through the phosphorylation cascade and plays a key role in regulating cell survival, differentiation, proliferation, migration, and apoptosis [33, 34]. There is evidence that PI3K/AKT inhibition effectively blocks pancreatic tumor progression. Our research revealed that the PI3K/AKT signaling pathway is significantly inhibited in the presence of linderalactone. Reversing the activation of the PI3K/AKT signaling pathway in pancreatic cancer cells may inhibit tumor activity. Therefore, we concluded that linderalactone may exert its activity by inhibiting the PI3K/AKT signaling pathway. We did not identify a direct target of linderalactone, so further study is needed.

In conclusion, our research shows that linderalactone has significant antitumor activity *in vivo* and *in vitro*. This compound inhibits tumor cell proliferation, migration, and invasion and induces cell cycle arrest and apoptosis through the PI3K/AKT signaling pathway. Our research might provide a novel therapeutic option for pancreatic cancer treatment.

## Data Availability

The research data used to support the findings of this study are available from the corresponding authors upon request.

## Supplementary Materials

Supplementary Materials Supplementary Figure 1: linderalactone inhibits the proliferation of pancreatic cancer cells. Supplementary Figure 2: linderalactone inhibits the migration and invasion of pancreatic cancer cells.
