# Supplementary material for: Linderalactone Suppresses Pancreatic Cancer Development In Vitro and In Vivo via Negatively Regulating PI3K/AKT Signaling Pathway
Source: J Oncol. 2022 Aug 4;2022:8675096. doi: 10.1155/2022/8675096 (PMC9371883; doi:10.1155/2022/8675096)
Supplement: Supplementary Materials — Supplementary Figure 1: linderalactone inhibits the proliferation of pancreatic cancer cells. Supplementary Figure 2: linderalactone inhibits the migration and invasion of pancreatic cancer cells. [file 8675096.f1.pdf]

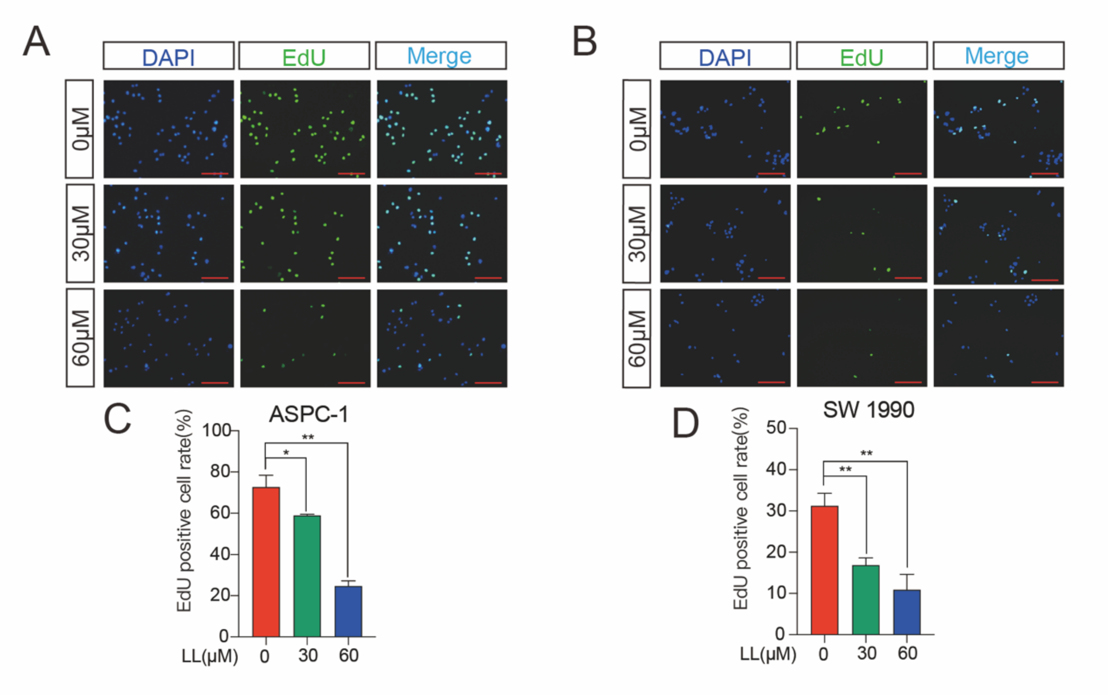

Supplementary figure 1 Linderalactone inhibits the proliferation of pancreatic cancer cells. A-B EdU staining eassay detected the proliferation of ASPC-1 and SW 1990 cell lines under varying concentrations of LL treatment after 24 hours. C-D Quantification of EdU stain: The percentage of EdU positive cells to the total number of cells. Scale bar = 200 μm. *$p<0.05$, **$p<0.01$.

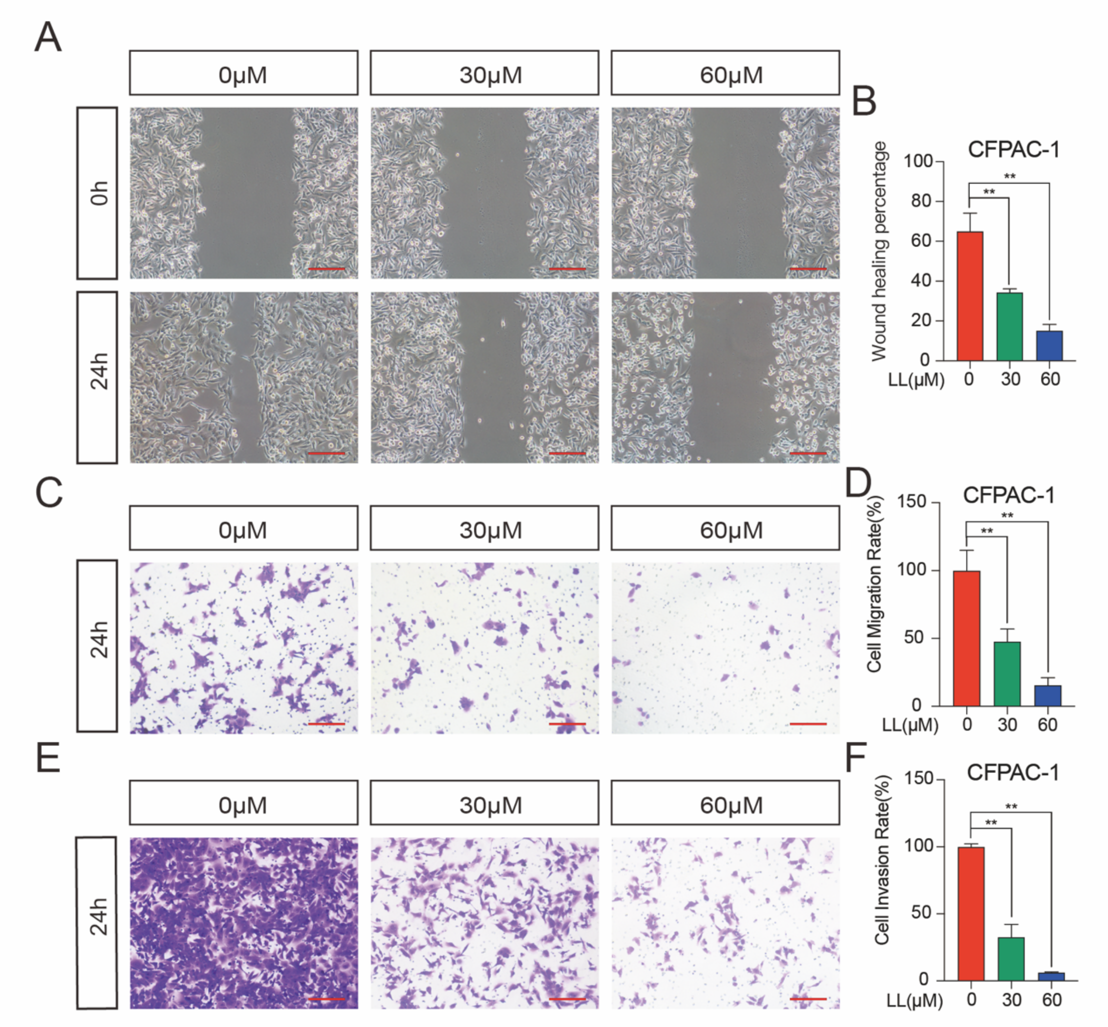

Supplementary figure 2 Linderalactone inhibits the migration and invasion of pancreatic cancer cells. A Wound healing assay detects the migration ability of CFPAC-1 under varying concentrations of LL after 24 hours. B Quantification of Wound healing assay: The area of cell migration in 24 hours accounts for the percentage of wound area in 0 hours. C The transwell assay detected the migration ability of cells under varying concentrations of LL treatment after 24 hours. D Quantification of transwell assay: percentage of compound treatment group in control group E Transwell assay with Matrigel to detect cell invasion ability under varying concentrations of LL treatment after 24 hours. F Quantification of transwell assay: percentage of compound treatment group in control group. Scale bar = 200 μm. *$p<0.05$, **$p<0.01$.
